# Clinical application of computerized evaluation and re-education biofeedback prototype for sensorimotor control of the hand in stroke patients

**DOI:** 10.1186/1743-0003-9-26

**Published:** 2012-05-09

**Authors:** Hsiu-Yun Hsu, Cheng-Feng Lin, Fong-Chin Su, Huan-Ting Kuo, Haw-Yen Chiu, Li-Chieh Kuo

**Affiliations:** 1 Department of Physical Medicine and Rehabilitation, National Cheng Kung University, Tainan, Taiwan; 2 Department of Physical Therapy, National Cheng Kung University, Tainan, Taiwan; 3 Department of Biomedical Engineering, National Cheng Kung University, Tainan, Taiwan; 4 Section of Plastic Surgery, Department of Surgery, National Cheng Kung University, Tainan, Taiwan; 5 Department of Occupational Therapy, National Cheng Kung University, 1 University Road, Tainan, 701, Taiwan; 6 Department of Physical Medicine and Rehabilitation, National Cheng Kung University Hospital, Tainan, Taiwan

**Keywords:** Sensation, Stroke, Sensorimotor control, Feedback control, Hand function

## Abstract

**Background:**

Hemianaesthesia patients usually exhibit awkward and inefficient finger movements of the affected hands. Conventionally, most interventions emphasize the improvement of motor deficits, but rarely address sensory capability and sensorimotor control following stroke. Thus it is critical for stroke patients with sensory problems to incorporate appropriate strategies for dealing with sensory impairment, into traditional hand function rehabilitation programs. In this study, we used a custom-designed computerized evaluation and re-education biofeedback (CERB) prototype to analyze hand grasp performances, and monitor the training effects on hand coordination for stroke patients with sensory disturbance and without motor deficiency.

**Methods:**

The CERB prototype was constructed to detect momentary pinch force modulation for 14 sub-acute and chronic stroke patients with sensory deficiency and 14 healthy controls. The other ten chronic stroke patients (ranges of stroke period: 6–60 months) were recruited to investigate the effects of 4-weeks computerized biofeedback treatments on the hand control ability. The biofeedback procedures provide visual and auditory cues to the participants when the interactive force of hand-to-object exceeded the target latitude in a pinch-up-holding task to trigger optimal motor strategy. Follow-up measurements were conducted one month after training. The hand sensibility, grip forces and results of hand functional tests were recorded and analyzed.

**Results:**

The affected hands of the 14 predominant sensory stroke patients exhibited statistically significant elevation in the magnitude of peak pinch force (p = 0.033) in pinching and lifting-up tasks, and poor results for hand function tests (p = 0.005) than sound hands did. In addition, the sound hands of patients were less efficient in force modulation (p = 0.009) than the hands of healthy subjects were. Training with the biofeedback system produced significant improvements in grip force modulation (p = 0.020) and better performances in the subtests of pin insertion (p = 0.019), and lifting of lightweight objects (p = 0.005).

**Conclusions:**

The CERB prototype can provide momentary and interactive information for quantitative assessing and re-educating force modulation appropriately for stroke patients with sensory deficits. Furthermore, the patients could transfer the learned strategy to improve hand function.

## Introduction

Stroke-related impairment often restricts patients from properly participating in the activities of daily living, and impedes social interactions. The stroke is judged to be the sixth most common cause of reduced disability-adjusted life years, which provides a common metric for meaningful comparison of the burden of risk factors, disease, and injury [1]. Motor deficits of the hands and upper extremities are important determinants, and strongly represent one’s ability to regain independence in daily and social living [2]. Previous reports describe that approximately 60–70% of stroke patients exhibit mild to severe hand dysfunction [2-5]; and up to 20% of stroke survivors were dependent in their basic daily living activities [6]. Such negative impacts on function are a strong motivation for researchers to investigate hand movement dynamics [7] and to develop effective therapeutic interventions for stroke patients [8].

Stroke patient hand dysfunction has been assessed by both quantitative and qualitative tests, such as the Brunnstrom recovery stages [9], maximum grip strength, the Fugl-Meyer assessment [10], and real-life Motor Activity Log questionnaires [11]. However, use of these traditional and common evaluation methods does not assess the delicate controls of the hand, such as spatio-temporal coordination of multi-segmented movements and precise force modulation. Investigating hand-to-object interactions by analysis of sets of time-series data obtained from joints of the upper extremities can provide a thorough understanding of the hand coordination of stroke patients [12]. During the past two decades, patient capacity for precision grip has become an indicator for investigating skilled movement control [13]. When an object is lifted and moved around in space, the pinch force should be modulated simultaneously with movement-induced load fluctuations [14-16]. The task-related sensorimotor processing depends on a complicated integration of feed-forward and feedback control mechanisms. Due to the decline in sensorimotor function suffered by stroke patients, affected hands do not have the ability to produce finger forces appropriate to an object’s manipulation [17,18]. A similar phenomenon was also observed in the sound hand of stroke patients [19]. However, motor dysfunction is generally considered as the main cause of hand function deficit; by contrast, less emphasis has been directed to the effects of sensory disturbances on functional performances even though approximately 50–85% stroke survivors with dysfunction of different sensory modalities have been reported [20].

Persons lacking sufficient ability for sensory modulation control generally use excessive force, or exercise clumsy manipulation when executing tasks [15,16,21-23]. Similar patterns occur for cerebral stroke patients with predominant sensory symptoms which indicate the patients with impaired sensibility still had sufficient hand motor functions, such as pinch and lift abilities [17,18,24], that is, improper programming of pinching behavior may lead to a clumsy hand in mild stroke patients. Thus, the purpose of sensory re-education is not only to provide opportunities to fulfill an individual’s sensory potential, but also to provide the chance to learn sensorimotor control strategies that are not emphasized in current clinical practices. Consequently, it is crucial for improving stroke patient quality of life to integrate suitable strategies for improving sensory function of the hand, and to promote useful sensorimotor operations into rehabilitation programs.

A better and more efficient motor response is learned using a well-integrated neurophysiological feedback system. However, most of the available biofeedback equipment and techniques currently available are used to retrain of movement and postural motor control [25,26]. Only few feedback trainings methods in the literatures have been suggested focusing on the break-up synergistic patterns and facilitation on poorly recruited muscles of elbow, wrist and finger extensors [27,28]. However, fine manipulative movements of thumb and fingers are difficult to regain, and it is challenging to monitor the outcomes via the feedback training. For those with hemiparesis post stroke, the ability to grasp may be an important indicator of the functional use of the upper extremities. A previous study found that biofeedback training provided significant restoration of grip force control in stroke patients [29]; training emphasis was placed on motor deficits and force production. Though awkward and inefficient finger movements of affected hands have been observed in hemianaesthesia patients [17,18,24], there was no literature mentioned on how to improve hand coordination caused by impaired sensibility. Therefore, we assumed that using of a system that can provide real-time and interactive information to patients with impaired sensation facilitates learning and execution at an appropriate functional level which offers a practical strategy for neuro-rehabilitation. According to the above-mentioned treatment framework, a control interface and software system, which linked up our previously designed pinch device [30,31] to set up a computerized biofeedback system was created for functional sensibility intervention in this study. The first specific aim of our study was to assess the pathophysiology of the clumsy hand movements of stroke patients with predominant sensory deficiency. We analyzed the relationship between the ability to modulate in a functional task and the actual hand capacity of the patients. The second aim was to investigate whether the traditional rehabilitation program, combined with computerized biofeedback training could improve the pinch coordination of chronic stroke patients with sensory deficiency. Additionally, we analyzed whether or not the beneficial effects on fine motor coordination were sustained at a one-month follow-up assessment.

## Methods

### Participants

In the first part of the study, we recruited fourteen stroke patients (nine males and five females) with predominant sensory symptoms to assess the pathophysiology of clumsy performance by the hands following a predominant sensory-stroke in the period March 2009 to September 2009. The predominant sensory symptoms refer to the patients of this study who suffered from impaired sensibility but still had sufficient hand pinch and lift ability. The subjects were referred by the Department of Physical Medicine and Rehabilitation, National Cheng Kung University Hospital. They should met the following criteria: (1) unilateral cerebral infarction, (2) CT scan or MRI imaging excluding pathologies other than unilateral cerebral hemisphere injury which was diagnosis by a physiatrist of physical medicine and rehabilitation, (3) had the ability to pinch and lift a pinch apparatus with their thumb and index finger, (4) were right handed. Exclusion criteria were as follows: (1) patients had major cognitive-perception deficit, i.e., praxis, memory, alertness and intellect which were screened by Mini-mental state examination, Lowenstein occupational therapy cognitive assessment (2) patients who could not understand or follow instructions. The affected hands included six dominant hands and eight non-dominant hands. The age range was from 26 to 74 years, and the mean age was 57.86 ± 12.03 years. An equal number of healthy, age-and-gender matched control participants, who were without evidence of any neurological deficit or orthopedic abnormality, were also recruited into this part. The second part of this study analyzed the effects of computerized biofeedback training for patients who should meet the criteria of the first part of the study. In addition, the probability for spontaneously regaining hand dexterity of the stroke patients should not be expected after 6 months of stroke onset [32]. To understand how the learned strategy through the prototype impacts on movement control of the affected hands, the essential requirement should be greater than 6 months post stroke onset. Ten subjects (seven males and three females) who fitted the requirements were recruited; the mean age was 57.1 ± 15.1 (age range: 26 to 74 years) years old. The mean time from stroke onset to our first evaluation was 18.8 ± 18.4 (duration range: 6–60) months. All participants were informed of the purpose of this study and signed consent forms approved by the Hospital’s Institutional Review Board.

### Instrumentation

#### Computerized Evaluation and Re-education Biofeedback (CERB) prototype

**Figure 1:**
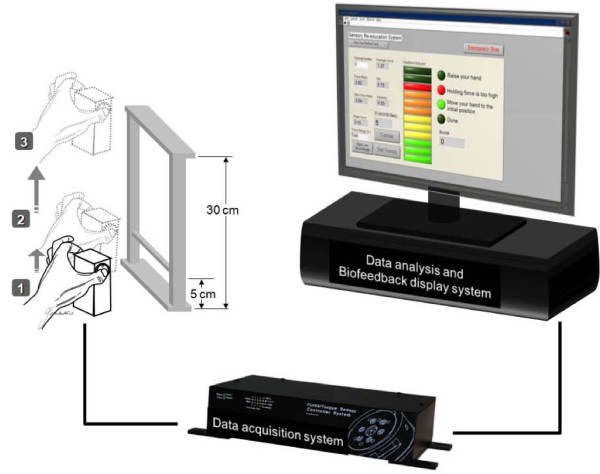
Schematic display of the computerized evaluation and re-education biofeedback (CERB) prototype.

The CERB prototype (Figure 1) comprised force-detection and force-feedback systems, which operated through the custom-built pinch apparatus (weight 480 g, dimensions 6.0 × 4.5 × 9 cm), and custom-made LabVIEW^TM^-based (National Instruments, Inc.) control interface, respectively. Participants pinched and lifted the pinch device, using the pulps of the thumb and index finger, to about 5 cm above the table, and held this position for 3 seconds. They then lifted the apparatus to a height of 30 cm and then slowly lower it to its initial position. Before formal data recording, participants were allowed three practice trials to ensure they completely understood this pinch-up-holding activity (PUHA) test. The data collection period for each trial was 15 seconds. The force parameters such as the peak pinch force (FP_peak_) during the lifting-up phase, the maximum load force (FL_max_) of the pinch apparatus at maximum upward acceleration onset during the lifting-up phase and the force ratio (the ratio of FP_peak_ to FL_max_) were collected and analyzed. A Matlab® (The MathWorks, Inc., Natick, MA) program was developed to find the FP_peak_, FL_max_ and force ratio in the pinch-up-holding task.

The computer interface consisted of a manual setting panel and real-time feedback display panel. Before the execution of the biofeedback intervention, the therapist should set up the target force range using the manual setting system. The target force range was selected to be in a range within the pinch apparatus loads up to 94% of the peak pinch force by the affected hand obtained during the initial evaluation. The 94% of peak pinch force value, was determined according to result of the average non-dominant hand producing 6% excessive force than the dominant hand to complete pinch-lifting task obtained in our previous study [30]. Based on the motor re-learning theory, motor planning of the affected hand would be more accurately programmed to optimize force during lifting through practice; therefore, we decided that this 6% difference could be possibly improved upon by the CERB prototype. Patients next performed new PUHA test trials in the training session. The detected force in the new test was compared with the target force level by a feedback loop. Once the force output fell outside the target range, visual and auditory cues were activated to inform the subject. Each participant received five repeated PUHA training runs in each treatment session. The exerted force and the force ratio between pinch force and load force were recorded, and displayed on the monitor in real time. The CERB protocol required 10–15 minutes per session, 3 times a week for 4 weeks.

#### Traditional sensory test

Traditional sensory evaluations including static and moving two-point discrimination (2PD) tests and the Semmes-Weinstein (SW) monofilament test were used to measure sensibility of the thumb pulp. The 2 PD test is a meaningful method of assessing tactile gnosis, while the SW monofilament test is used to measure the cutaneous pressure threshold. To understand the ability of sensing joints movement of patients, the proprioception test was set patient’s sound arm into a specific position then patient is asked to place their sound arm into the same position.

#### Hand function test

In the study, we used two timed measure of hand function tests. The Purdue pegboard test was used to test the gross movement of arm, and fine fingertip dexterity [33]. Subjects were asked to pick and place as many pins as possible into the holes in thirty seconds. To understand the ability of adapting the manipulative forces in executing functional task, subtests 6 and 7 (lifting an empty tin can and a full tin can weighing 1 lb, respectively) of the Jebsen-Taylor Hand Function Test were used as force –matching tasks to assess how peripheral sensory information contributed to hand function [34]. Each of the tests is the widely used as an outcome measure for hand functions.

### Experimental procedures

All subjects received their initial evaluations by recording basic personal information, the force parameters obtained from a PUHA test, results of the Purdue pegboard test, the Jebsen-Taylor hand function test, and traditional sensibility tests. Participants in the intervention group received a 25-minute traditional rehabilitation program (including task-based trainings by functional activities, inhibition techniques for normalizing the abnormal tone and training with daily living skills), in addition to the ten-minutes biofeedback training over 12 successive sessions, which were conducted three times per week and supervised by an occupational therapist. After undergoing this 4-week program, tests carried out in the initial evaluations were repeated for comparison purposes. Follow-up measurements were conducted one-month post-training.

### Data analysis

We analyzed precision pinch ability of stroke patients with predominant sensory deficits for improvement of hand function resulting from the computerized biofeedback training. The precision grip parameters were the peak pinch force (FP_peak_), and the FP_peak_ and FL_max_ force ratio achieved during the PUHA test.

The Wilcoxon signed ranks test was used to test the differences in pinch force adjustment parameters obtained in the PUHA task and hand function tests, for the stroke patients’ normal and affected hands. The Wilcoxon signed ranks test was also used to analyze the differences in the magnitude of force ratio between the sound hand of stroke patients and the matched-hand of healthy control subjects. The Pearson correlation test was used to determine the correlation between tactile sensibility and pinch force adjustment in the PUHA task, and the hand function test in stroke patients. To understand the training effect of CERB prototype, the Friedman’s test was used to analyze the responsibility of pinch force modulation and results of hand function test for the stroke patients before, after and 1-month’s follow-up completing computerized biofeedback training.

## Results

**Figure 2:**
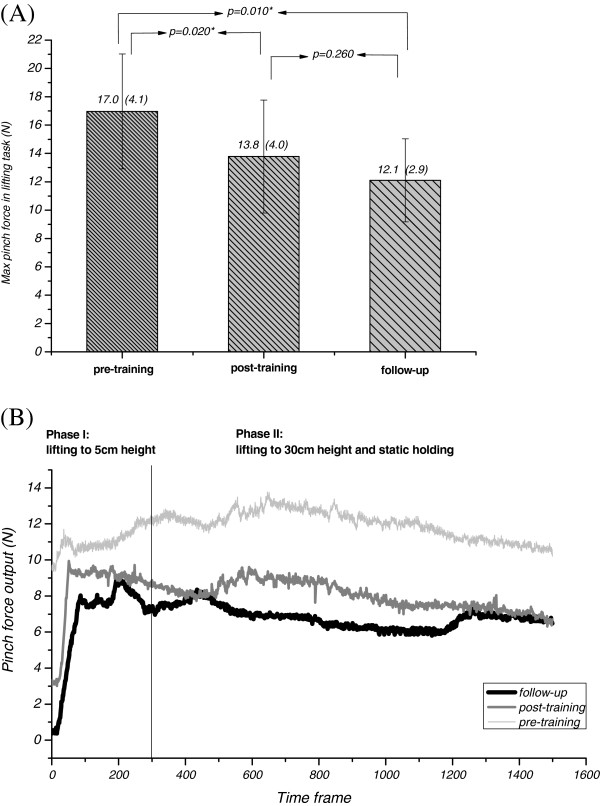
The improvements in the grip force modulation after computerized force reeducation, (A) The obtained peak pinch force at different time-point, (B) The pinch-force output with time series before training, after training and at the follow-up assessment of subject 5.

The sensory functions of 14 stroke patients are provided in Table 1. All subjects in the experimental group had tactile sensibility impairment. These patients exhibited clumsy performances in either the PUHA task or the hand function tests for the affected hands. The differences in detected force parameters between the sound and affected hands that were obtained from the PUHA and hand function tests are shown in Table 2. Most force and functional parameters reveal statistically significant differences (p < 0.05) between the affected and sound hands, except for difference in FP_peak_ (p = 0.055), which is close to 5% statistical significance level. The data in Table 3 indicates a moderate correlation between tactile ability, and parameters regarding force interaction for hand-to-object, and hand function tests with statistical significance (p ≤ 0.05). To understand the ipsilateral contributions to sensorimotor control, we analyzed the sound hand’s force modulation ability. The ability of stroke patients to produce finger forces for object manipulation using the sound hand was less efficient than it was for the healthy subjects. The mean force ratio was 3.36 in the sound hands of stroke patients, while healthy subjects exhibited a ratio of only 2.62 (p = 0.011*). In the second part of the experiment, ten patients who received the biofeedback intervention showed significant improvements in grip force modulation and ability to control force after training, and in the one-month follow-up assessment (Figure 2A, B). From actual hand manipulation perspectives, patients produced better performance in the subtests of pin insertion and lifting a lightweight object after training (Table 4). Peak force ratio improved significantly from 3.54 to 2.97 (p = 0.037*) after receiving training, and the effect was still apparent at the one-month follow-up (2.69 ± 0.52). The difference in force-ratio after-training, and at the one-month follow up was insignificant (p = 0.386).

**Table 1:** Sensory status of recruited subjects

	Proprioception of shoulder and elbow	Proprioception of hand	*Classification of 2PD sensibility	**Scale of interpretation of SW test
P1	impaired	loss	S1	Loss of protective sensation
P2	intact	intact	S4	Diminished light touch
P3	intact	intact	S4	Diminished light touch
P4	intact	intact	S4	Diminished light touch
P5	loss	loss	S1	Loss of protective sensation
P6	intact	intact	S4	Diminished light touch
P7	impaired	impaired	S3+	Loss of protective sensation
P8	intact	impaired	S3+	Diminished protective sensation
P9	intact	loss	S1	Loss of protective sensation
P10	intact	intact	S3+	Diminished light touch
P11	intact	intact	S4	Diminished light touch
P12	intact	intact	S4	Diminished light touch
P13	intact	loss	S3+	Diminished protective sensation
P14	intact	intact	S3+	Diminished light touch

*: S1: recovery of deep pain; S3^+^: has 2PD 7–15 mm range; S4: includes 2PD 2–6 mm range [35].

**: [36].

**Table 2:** The difference of force parameters and hand function between the affected and sound hand of stroke patients (n = 14)

Parameters	Affected hand Mean (SD)	Sound hand Mean (SD)	*P*-value
Force ratio: FP_Peak_/FL_max_	3.96 (1.13)	3.36 (0.77)	0.033*
Peak Pinch force: FP_peak_ (N)	18.81(5.2)	16.12 (3.77)	0.055
Purdue test	6.35 (4.51)	12.31 (2.76)	0.002*
Jebsen-Taylor lifting light object (secs)	7.89 (3.62)	4.40 (1.16)	0.001*
Jebsen-Taylor lifting heavy object (secs)	7.40 (3.17)	5.16 (1.77)	0.001*

Statistics: Wilcoxon signed ranks test; significance level was set at 0.05.

**Table 3:** Correlation between force parameters, hand function tests and pressure threshold test in a lifting performance

Parameters		Force ratio (Max)	Pinch force (Max)	Purdue test	Time (lifting light objects)	Time (lifting heavy objects)
S-W (pressure threshold test)	r	.576	.562	−.564	.709	.552
	*p*-value	0.031*	0.036*	0.036*	0.005*	0.041*

**Table 4:** Training effects of computerized force re-education on finger dexterity

	Pre-training Mean (SD)	Post-training Mean (SD)	Follow-up Mean (SD)	*p*-value
Inserting pins (Numbers)	6.4 (5.1)	7. 6(5.1)	8.1(5.7)	0.019*
Time of lifting light objects (seconds)	8.05(4.01)	6.71(4.24)	6.65 (4.09)	.005*
Time of lifting heavy objects (seconds)	7.41(3.50)	6.67(3.18)	6.45 (3.33)	.142

## Discussion

Sensory-related deficits are mentioned less often than motor impairment is, when discussing the rehabilitation outcome of stroke patients. However, some researchers have proposed that insufficient sensory information contributes to impaired hand function [15,37]. In this study, 100% of the patients with impaired-to-loss pressure threshold could not response to stimulation accurately and immediately; eight (57%) of them with impaired pressure threshold also exhibited diminished discriminative sensation, that is, the patients could not recognize objects which they were holding; and six (42.8%) of them with impaired-to-loss proprioception of the shoulder and elbow were unable to sense movement of joints when moving properly. Though the motor function was unaffected of the recruited patients, the impaired sensory function resulted in inefficient force interaction between fingers and object so much as overall hand functions of the patients (Table 2). The force parameters acquired during the pinch task indicate that the subject’s sound hands perform with greater force-control precision in functional tasks, and produce better results in the hand function test than the affected hands do, and previous studies produced similar results [2,17,18]. In recent years, there was a growing literature exploring how the sensory disturbance leaded to motor symptoms and clumsy hand function [34,38]. Insufficient sensory signals from mechanoreceptors of digits making the patient unable to judge the characters of the object timely, such as: shape, texture and weight [39]; therefore, elevation of pinch force is a compensatory strategy used by patients with impaired sensibility to prevent an object from slipping during execution of a functional task [40]. Results of a recent longitudinal study revealed that a strong relationship between finger force control ability and moving 2PD tests and SW monofilament test (correlation coefficient was 0.810 and 0.852 respectively) for the peripheral nerve injury patients [41]. Similar finding was obtained in this study, we observed a statistically significant moderate and quantitative correlation between the ability to exercise force control, and tactile threshold and a similar correlation occurred between hand function and tactile threshold. In particular, a significantly high correlation between tactile sensibility and the time required to lift light objects demonstrated that subjects needed considerable time to adapt exertion force to lifting a lightweight object, when they were unable to acquire sufficient sensory information about the objects’ physical characteristics.

The finger-force modulation PUHA task results for sound hand of the subjects with sensory pre-dominant stroke also showed greater inefficiency than results for healthy control subjects did. A few researchers reported similar findings, using different experimental models [18,19]. Motor deficits are often evident on the side of the body that is contralateral to the side with brain damage following stroke, and the inter-limb coupling mechanism might contribute to the subtle impairment seen on the sound side [42,43]. Stroke patients receiving traditional assessments for evaluation of stroke patient hand function demands concentration while performing the motor tasks, and unfortunately such assessments cannot always detect the true abilities of the sound hand [44]. While the coupling of pinch force modulation, with changes in loading is an automated response, it is not subject to the examinee’s conscious control [45]. Therefore, reduced motor control efficiency is a potential indicator of sound hand function, because of reduced sensorimotor control, though in the present study, motor control efficiency was unaffected by attention or cognitive deficits. That is to say, detection of the hand-to-object force interaction could be a valid and reliable tool for testing the sound hand function of stroke patients, and could be an indicator of the ability to participate in daily living activities.

In the second part of the study, all the computerized force re-education subjects were in the chronic phase (post-event duration was greater than 6 months). Though a lot of literatures proved that the probability of regaining upper extremity function should not be expected at 3–6 months after stroke onset [3,32]; nevertheless, we found that administering the computerized biofeedback intervention could improve pinch force modulation for stroke patients with sensory deficiencies. One previous study proposed a novel tracking system for the assessment and training of grip force control in a patient with traumatic brain injury; the authors found the patient gained improvements in grip force control after training with the tracking system [29]. However, the patient’s sensory condition and hand manipulation ability had not been quantitatively analyzed in their series of investigations. In our study, subjects exhibited a noticeable training effect, not only on the pinch force modulation, but also in hand coordination, and still showed these gains one month after completing the computerized biofeedback and re-education program. Thus, the sensorimotor retraining program was successful in improving the subjects’ functional performances. The improvements seen in the hand function test following completion of the training program meant that patients had learned to modify their pinch strategy to meet the demands of the task, and so facilitate movement control of their hand. This study’s results support the hypothesis that a learned strategy for control of pinch force can promote hand coordination of stroke patients in functional tests. The biofeedback re-education program was suited for providing threshold adjustments, monitoring, and real-time feedback to aid the restoration of deficient sensorimotor controls. The CERB prototype could be used as an enrichment of existing sensorimotor therapy, to enhance the function of an affected hand, especially in the Purdue pegboard pin insertion test, and the Jebson Taylor lightweight object lifting task subtests. The experimental evidence indicates that meaningful sensorimotor retraining should commence following a stroke to improve functional use of the affected hand, and our novel bio-interface feedback system is an effective therapy device for such hand function retraining.

This report introduced a system that can provide real-time and interactive information for quantitative assessing and re-educating the delicate hand function for stroke patients with sensory deficits. Though the numbers of recruited subjects were relatively small, we demonstrated that there exists a direct correlation between the measured application force and actual hand function. In addition, the obtained results of the study showed strong evidence that the benefit of using CERB prototype in stroke rehabilitation is not only improving the ability to modulate force appropriately and the learned strategy also could be transferred to improve hand functions. Furthermore, the effectiveness of treatment could be maintained for one month after completing the treatment. Thus, we conclude that the proposed conjunct system in this study are suited for evaluating and restoring sensorimotor function for patients with impaired sensibility even for chronic stage patients. In future work, we intend to investigate long-term effects of biofeedback training on hand function for patients with sensorimotor dysfunction.

## Competing interests

The authors declare that they have no competing interests of this study.

## Authors’ contributions

HYH, HYC and LCK are the main contributors in the study design, data collection and conventional sensory assessments. FCS carried out the control and technical problem solving of the Evaluation and Biofeedback System. HTK participated in the registration and physical examinations for the stroke patients. HYH, CFL, FCS and LCK participated in the data interpretation, analysis and interpretations. HYH, CFL and LCK helped to draft the manuscript. All authors read and approved the contents and format of the final manuscript.
